# Development of approach to an automated acquisition of static street view images using transformer architecture for analysis of Building characteristics

**DOI:** 10.1038/s41598-025-14786-3

**Published:** 2025-08-08

**Authors:** Seunghyeon Wang

**Affiliations:** Institute for Environmental Design and Engineering, University College London, London, WC1H 0NN UK

**Keywords:** Static street view images, Google street view, Building characteristics, Image augmentation, Deep learning, Convolutional neural network, Civil engineering, Scientific data

## Abstract

Static Street View Images (SSVIs) are widely used in urban studies to analyze building characteristics. Typically, camera parameters such as pitch and heading need precise adjustments to clearly capture these features. However, system errors during image acquisition frequently result in unusable images. Although manual filtering is commonly utilized to address this problem, it is labor-intensive and inefficient, and automated solutions have not been thoroughly investigated. This research introduces a deep-learning-based automated classification framework designed for two specific tasks: (1) analyzing entire building façades and (2) examining first-story façades. Five transformer-based architectures—Swin Transformer, ViT, PVT, MobileViT, and Axial Transformer—were systematically evaluated, resulting in the generation of 1,026 distinct models through various combinations of architectures and hyperparameters. Among these, the Swin Transformer demonstrated the highest performance, achieving an F1 score of 90.15% and accuracy of 91.72% for whole-building façade analysis, and an F1 score of 89.72% and accuracy of 92.27% for first-story façade analysis. Transformer-based models consistently outperformed 810 CNN-based models, offering efficient processing speeds of 0.022 s per image. However, differences in performance among most models were not statistically significant. Finally, this research discusses the practical implications and applications of these findings in urban studies.

**Supplementary Information:**

The online version contains supplementary material available at 10.1038/s41598-025-14786-3.

## Introduction

Static Street View Images (SSVIs) capture detailed visual information of building façades, including architectural features such as window types, exterior cladding materials, and the overall façade design. Each SSVI is georeferenced, linking images directly with precise latitude and longitude coordinates^1^. Additionally, these images include camera parameters such as heading, pitch, and zoom, allowing users to view and analyze buildings from various angles. One prominent example of an SSVI platform widely used for building façade analysis is Google Street View (GSV)^2^.

Utilizing SSVIs for building façade analysis offers an efficient and cost-effective alternative to traditional, labor-intensive on-site surveys. For instance, GSV covers more than 16 million kilometers and is accessible in 83 countries^3^. Such extensive coverage facilitates large-scale building analyses relevant to urban planning, nationwide building surveys, and architectural studies.

Many studies have extensively leveraged SSVIs for various tasks, employing diverse deep-learning techniques ranging from basic classification methods to segmentation and object detection approaches. Specific applications include building usage classification^4,5^ façade material recognition^6–8^ building type identification^3,9^ floor-level and story estimation^10–12^ building age determination^13–15^ detection of façade deterioration^16^ identification of façade elements such as windows and walls^17^ and segmentation of tile peeling on building façades^18^. Additionally, urban feature mapping tasks, such as pole and streetlight detection^19^ and urban form analysis^20–22^ have significantly benefited from the application of SSVIs.

**Fig. 1:**
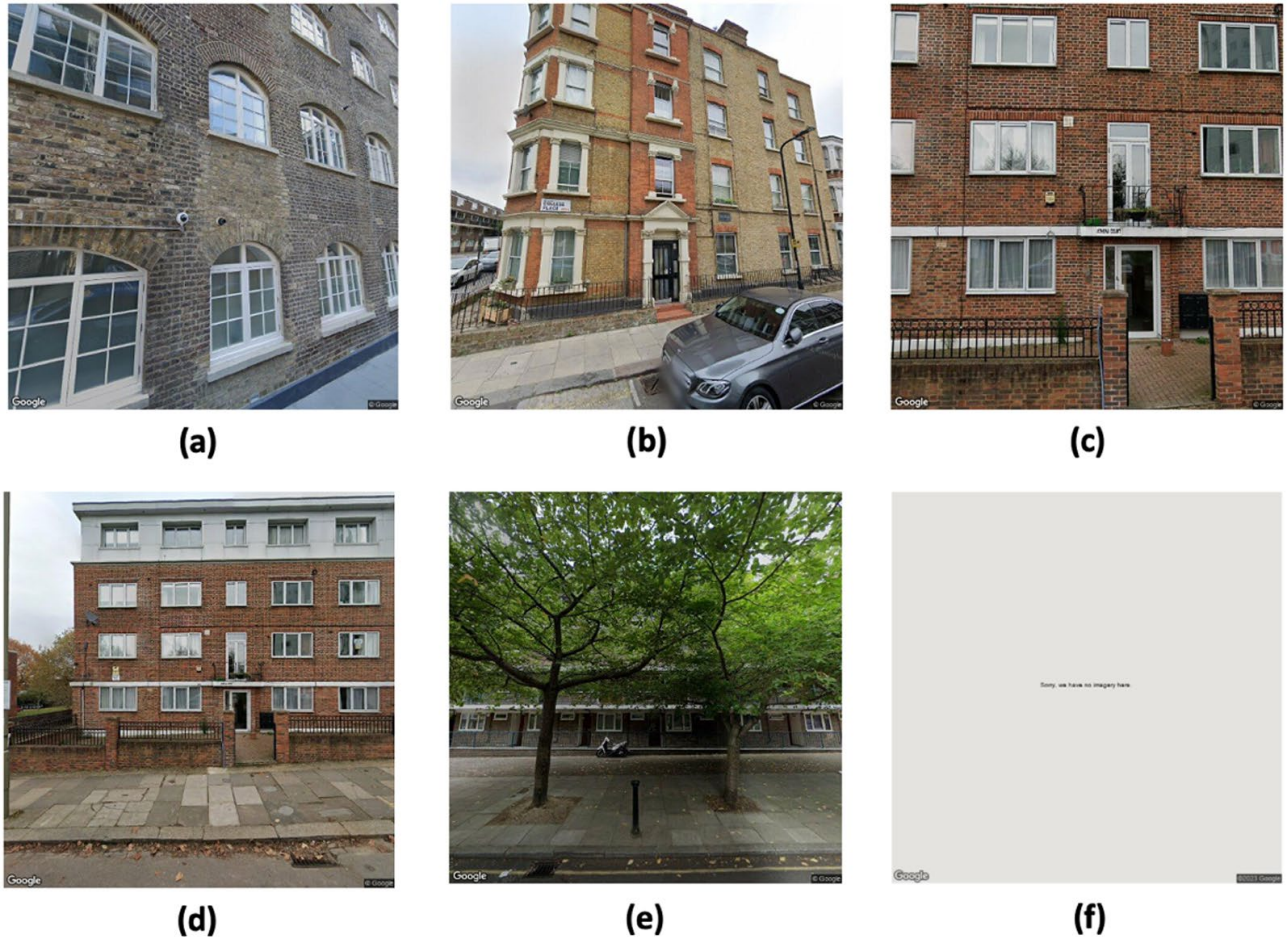
Examples of the three types of SSVIs.

Despite advantages of SSVIs, not all images of building facades are suitable for analysis. In terms of the practical application of these images, the acquired SSVIs of buildings can be categorised into the following three types:

(1) Usable images: These are images in which visual cues for specific tasks are clearly visible. For instance, exterior cladding materials (e.g. wood, and brick) can be interpreted by observing the evenly magnified images of building façade, as shown in Fig. 1a. However, images that display the entire façade of a building are crucial for estimating the number of storeies, as illustrated in Fig. 1b.

(2) Potential images: The visual cues in these images might not be immediately apparent. Nevertheless, by adjusting the camera parameters at the same address, they can potentially become usable images. For instance, Fig. 1c and d were obtained from the same address but with different camera parameters. Figure 1c is more zoomed in than Fig. 1d, making tasks that require a view of the entire building façade, such as estimating the number of storeies, impossible. However, by zooming out using various camera settings, the degree of clarity needed to determine the number of storeies can be obtained, as shown in Fig. 1d.

(3) Non-usable images: These images are unsuitable for analysis, even after adjusting camera parameters. A frequent issue is the obstruction of substantial portions of the building façades by external objects, such as trees or fences, as illustrated in Fig. 1e. Additionally, certain addresses do not have available SSVIs, resulting in error messages as shown in Fig. 1f.

Identifying and differentiating between usable, potential, and non-usable images currently relies heavily on manual interpretation. Furthermore, as no universal camera parameter settings are suitable for every building^10^ manual adjustments are often required, especially for converting potential images into usable ones. This manual approach is labor-intensive and inefficient, particularly when surveying extensive geographic regions.

Despite the broad utilization and proven effectiveness of SSVIs across various fields, the reliance on manual adjustments highlights a significant research gap in automating the acquisition and classification of suitable SSVIs. Advanced supervised deep learning methods, notably convolutional neural networks (CNNs), have shown strong performance in tasks utilizing SSVIs^23–25^. Recently, transformer-based models have emerged as powerful deep learning architectures due to their superior ability to capture long-range dependencies and contextual information within images, often outperforming traditional CNN-based methods^26–30^.

This study addresses the identified research gap by developing automated transformer-based methods specifically designed for acquiring and classifying SSVIs. It aims to automatically identify images suitable for two key analysis tasks: assessing entire building façades and detailed inspection of first-story façades. The primary contributions of this research include:

1. A total of 1,026 models were developed by combining five transformer-based architectures with various hyperparameter settings, representing the first automated approach for SSVI acquisition and classification.
2. Two specific image analysis tasks were targeted: whole-building façade images and first-story façade images.
3. A comprehensive comparative analysis was performed to evaluate the effectiveness of advanced transformer-based architectures against 810 traditional CNN-based models in classifying SSVIs.
4. Different model evaluation metrics were employed, including analyses of performance variation, Grad-CAM visualization, impacts of varying image conditions, and paired bootstrap statistical testing.

## Proposed approach

**Fig. 2:**
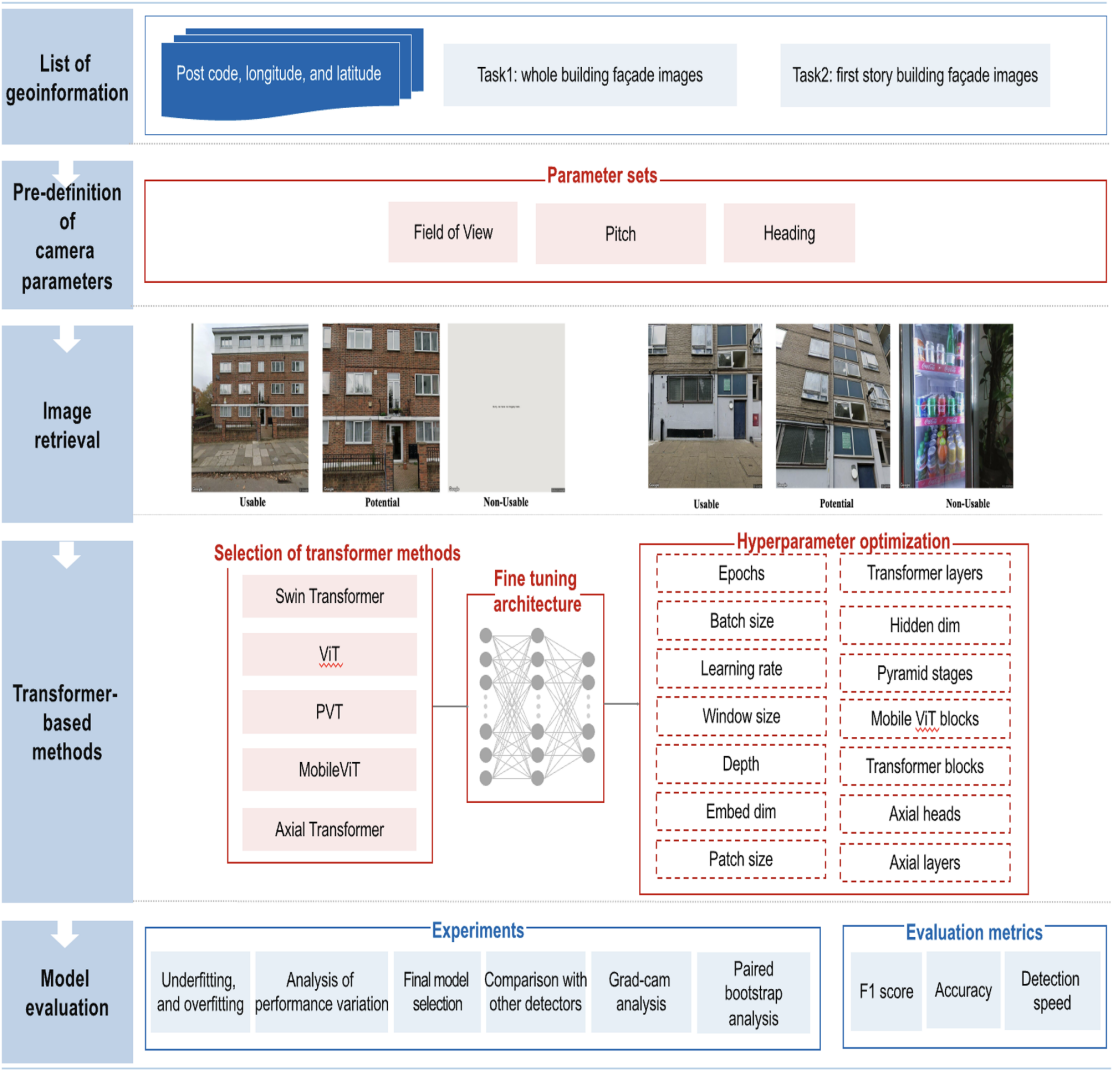
Workflow for the demonstration of proposed method.

In order to enable usable SSVIs to be quickly and accurately collected, an overview of the workflow, highlighting the approach used and its main procedures, is illustrated in Fig. 2. The process involves the following five key steps:

1) List of geoinformation: based on a specified area of interest, a list of geoinformation such as building addresses, and spatial information (e.g. latitude and longitude) is created.

2) Pre-definition of camera parameters: the ranges of the camera parameters used, such as pitch, and heading, are randomly defined, and assigned in advance.

3) Image retrieval: based on the list of remaining addresses, and one of the sets of parameters, the images are retrieved from the SSVI platform.

4) Transformer-based classification: a transformer-based model is used to classify the retrieved images into usable, potential, and non-usable images. Addresses linked to both usable and non-usable images are then discarded from the list.

Following the initial steps outlined above, the processes of image retrieval and classification using deep learning techniques are iteratively conducted on additional candidate images. This iterative approach continues until all predefined sets of camera parameters had been thoroughly examined. Consequently, the resulting dataset comprises exclusively high-quality images that clearly depict specific attributes of buildings, each accurately associated with relevant geographic metadata. Finally, multiple analytical approaches and diverse performance metrics are employed to rigorously evaluate the effectiveness of the implemented models.

### Visual cues for usable images

**Fig. 3:**
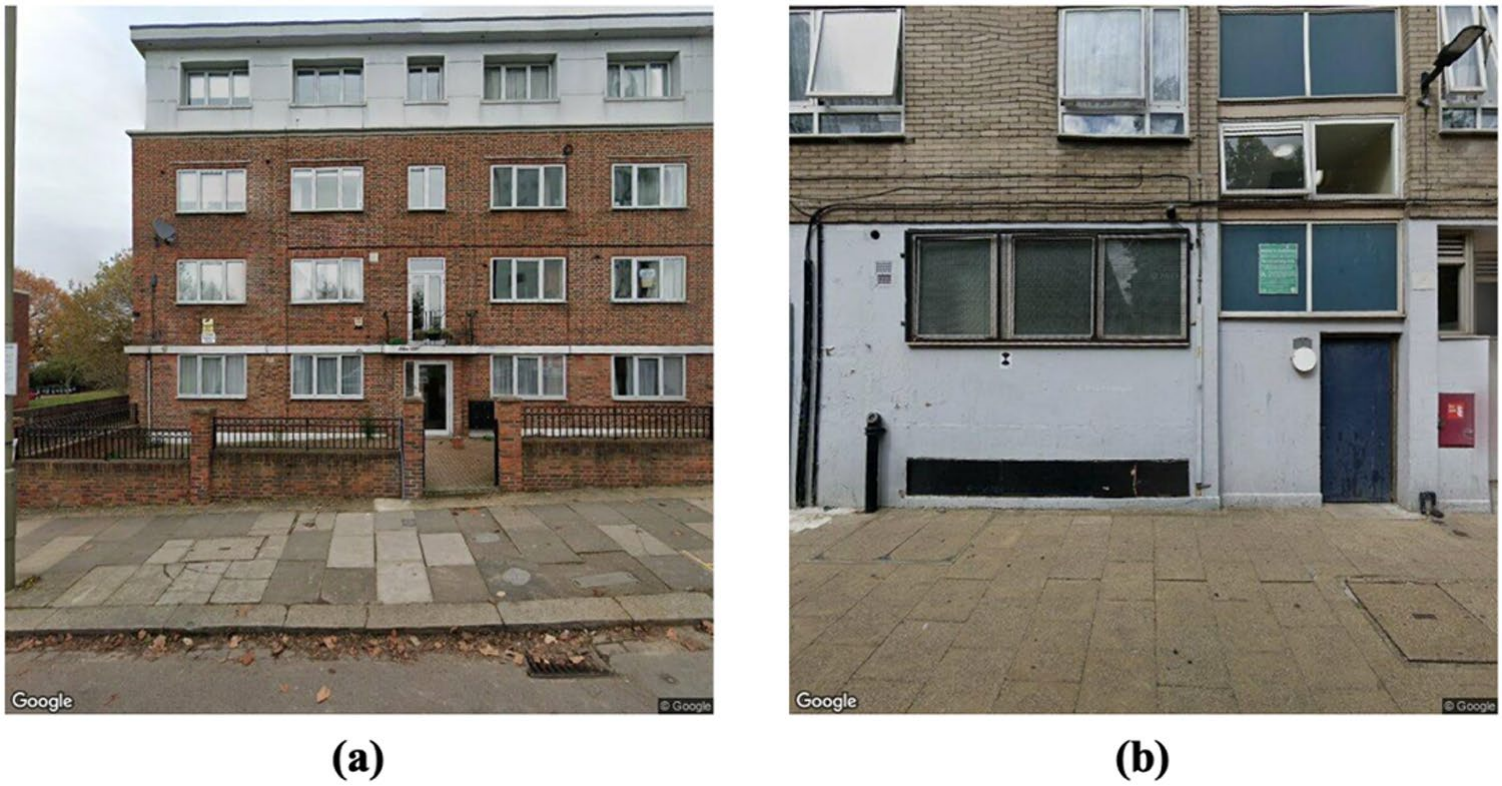
Examples of target building façade images for each task.

The selection of usable SSVIs varies based on the specific requirements of different analytical tasks. An image that is ideal for one type of building analysis might be unsuitable for another. For instance, images that clearly capture an entire building’s façade are essential when evaluating attributes of window wall ratio or determining the number of stories in a building, as illustrated in Fig. 3a. In contrast, for tasks such as identifying the presence of parking lots, images that primarily showcase the first story of the building may be sufficient, as depicted in Fig. 3b. This research focuses on two types of images: those that capture the entire building façade; and those that mainly show the first story of the building.

### Transformer-based image classification

Transformer-based image classification generally involves two primary components: a feature extractor and a classifier. The feature extractor generates pertinent feature maps from an input image, while the classifier utilizes these features to categorize the image into predefined classes.

Robust backbone architectures are frequently adopted due to their demonstrated effectiveness across diverse image classification tasks^31^. These architectures exhibit distinct trade-offs between accuracy and computational efficiency, prompting exploratory analyses to identify the most suitable model for accurate yet efficient classification.

Transformer-based models often surpass CNN architectures in accuracy by capturing intricate image relationships. However, this improvement commonly comes with increased computational demands, posing challenges for real-time applications^28,32^. Tasks like safety helmet monitoring—characterized by small objects, occlusions, and varying lighting—highlight the importance of selecting models that balance accuracy and efficiency^33^. Therefore, this study evaluates five advanced transformer-based architectures, summarized succinctly in Table 1.

**Table 1:** Summary of key characteristics for each backbone architecture.

Aspect	ViT	Swin Transformer	PVT	MobileViT	Axial Transformer
Core concept	Pure transformer	Hierarchical transformer	Pyramid transformer	Hybrid convolution-transformer	Axial decomposition attention
Feature extraction	Global context features	Multi-scale hierarchical features	Multi-scale pyramid features	Balanced local-global features	Axial long-range dependencies
Attention Complexity	Quadratic (with image patches)	Linear scaling (with image size)	Efficient spatial-reduction (linear)	Optimized for edge devices (linear)	Linear scaling (axial decomposition)
Resource Demand	High	Moderate	Moderate	Low	Moderate

### Classifier

Following the feature extraction process, a FC layer is attached. The node dimensionality of the input layer matches the size of the extracted features to enable it to receive them as input. The number of neurons in the output layer is adjusted to correspond to the number of classes; in this case, three: usable images, potential images, and non-usable images. The architecture of hidden layers can vary considerably, and optimizing their design is typically considered a form of hyperparameter tuning^34,35^. In this study, the classifier consists of two hidden layers comprising 32 and 16 nodes, respectively.

### Model optimization process

#### Transfer learning

Training deep learning architectures from scratch often requires significant computational resources, considerable training time, and large amounts of labeled data. To overcome these challenges, pre-trained models—previously trained on extensive datasets—can serve as efficient alternatives^36^. Such models utilize features learned from prior training to effectively accomplish diverse tasks. In this research, pre-training was performed using ImageNet, a prominent benchmark dataset extensively adopted in computer vision research. ImageNet^37^ is a comprehensive repository comprising over 14 million labeled images across a broad spectrum of categories, including general object classes such as buildings and people. Therefore, the feature extraction components of the transformer architectures employed in this study were initialized using pre-trained weights derived from the ImageNet dataset.

#### Image augmentation techniques

The development of datasets for deep learning purposes often requires significant effort and time. To mitigate this, the practice of image augmentation is employed, which effectively increases the size of artificial training datasets. This study incorporates six distinct augmentation techniques which are tailored to each task. The rationale behind each technique is concisely articulated herein, and a full explanation, replete with mathematical details, is available in prior research^29,38^.

Given that SSVIs are captured outdoors, variations in lighting due to environmental factors such as the weather or time of day are common. Brightness augmentation is used to simulate these conditions by altering the light intensity in the images, resulting in variations from brighter to darker representations^39^. Contrast augmentation is another technique that can improve the visibility of images by enhancing the luminance contrast, thereby making the defining features of objects within the image more pronounced, which is especially useful for highlighting the contours and edges of architectural features^40,41^. To accommodate the different angles from which images are taken, perspective transformation augmentation is applied to adjusts the image’s homography matrix. This ensures that the model can accurately recognise buildings regardless of the angle from which they were photographed. Similarly, scale augmentation, which is a type of affine transformation, replicates the different perspectives that result from varying distances to the buildings, and takes into account the actual disparities between them in terms of size.

Images of building façades may sometimes exhibit a lack of vertical alignment, which results from the camera not being level during image capture. This can give the impression that buildings are slanted. Rotation augmentation corrects this by enabling the accurate determination of building characteristics, such as typology and story count, irrespective of the angle at which the building is presented in the image. Finally, shear augmentation addresses the visual distortion that occurs when the camera is not perpendicular to the building façade, causing buildings to look as if they are tilting to one side. This geometric transformation skews the image along a particular axis to simulate this effect^42^.

#### Optimization of the hyperparameters

Selecting suitable hyperparameters is crucial for maximizing the performance of transformer-based models; however, performing exhaustive hyperparameter optimization typically requires substantial computational effort^6,43^. A practical solution is to define hyperparameter ranges informed by prior research and empirical knowledge. Therefore, hyperparameter ranges and specific values were thoughtfully determined, enabling the exploration of diverse configurations for transformer-based approaches. Table S.1 outlines these hyperparameters clearly, differentiating between common parameters applicable to all methods and those unique to individual methods, along with the total number of model configurations evaluated.

### Evaluation of model performance

#### F1 score, and accuracy

In classification tasks, metrics such as the F1 score and accuracy are commonly utilized to evaluate model performance. The F1 score combines precision and recall into one metric, effectively balancing the model’s capability to accurately detect actual detections (recall) with its ability to minimize incorrect detections (precision). A detailed explanation of the F1 score is provided by^44^ A high F1 score signifies that the model accurately identifies faults with minimal false alarms and missed detections, which is critical for ensuring reliable system operation. Accuracy, on the other hand, calculates the proportion of correctly classified instances relative to the total number of evaluated instances, offering a direct measure of the model’s overall correctness^45^.

#### Detection speed

The detection speed is the time computed by a model for a single frame. The speed is measured in Frames Per Second (FPS). A key goal of assessing detection speed in this research is to identify whether certain deep learning models, while maintaining equivalent accuracy, can achieve a faster performance due to different architectural designs, thereby making them more appealing.

## Experiment

### Dataset preparation

#### Original dataset

**Fig. 4:**
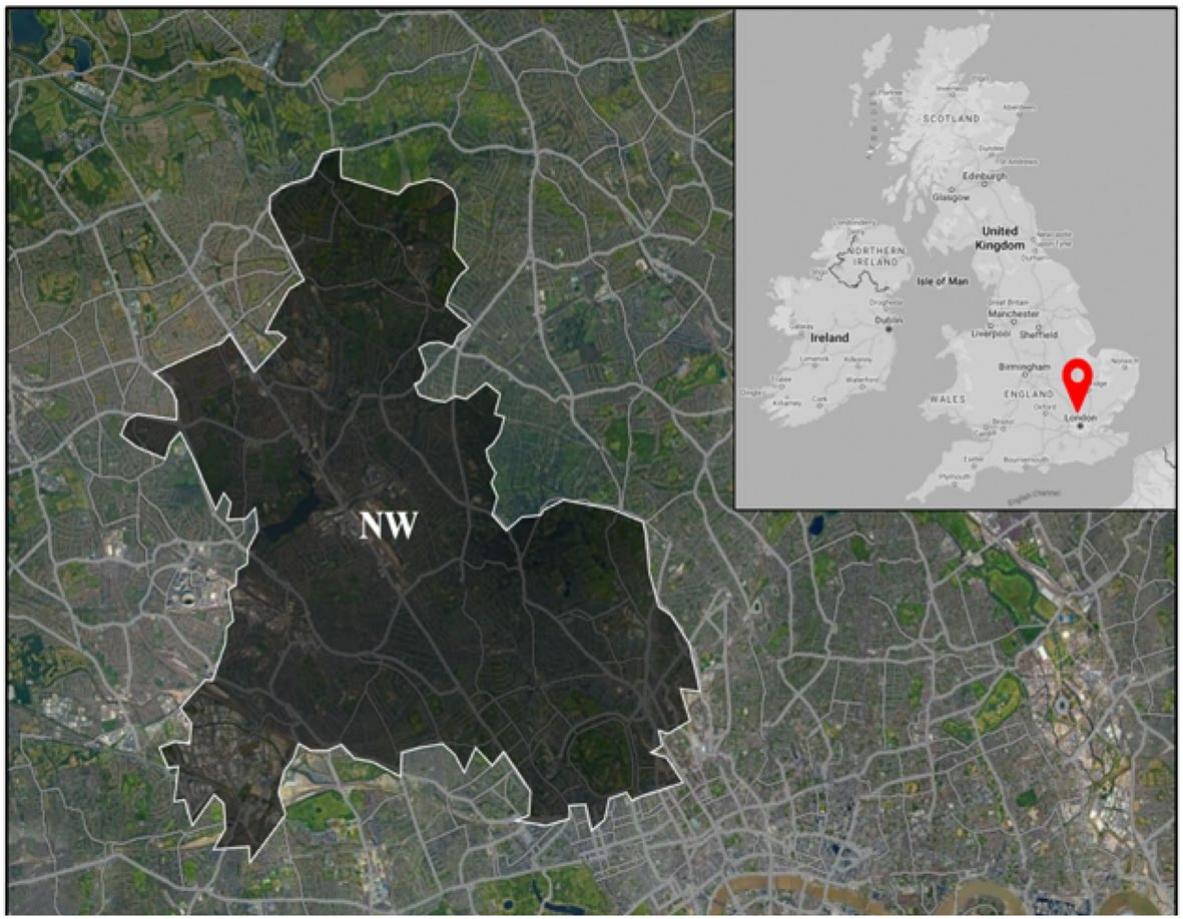
Area of NW London used for data collection.

For the case study, the city of London in the UK was selected due to it being legally permissible to collect the SSVIs. A random selection of addresses for 1,000 buildings was made from the North-West (NW) area of London, as depicted in Fig. 4, using the OS Data Hub, which is Great Britain’s national mapping agency (https://osdatahub.os.uk/). These addresses were then used to retrieve and download corresponding building images via the GSV Static API (https://developers.google.com/maps/documentation/streetview/intro).

Within the API, three critical camera parameters were adjusted because they substantially affected the visual cues necessary for the clear identification of building characteristics. The parameters include: the Field of View (FOV), which sets the zoom level or the breadth of the scene visible; pitch, which defines the camera’s vertical tilt relative to the street view vehicle; and heading, which determines the camera’s lateral orientation. These parameters have been shown to be significant in prior research^10,11^. The optimal ranges for these parameters were established through preliminary trials and adjustments.

**Fig. 5:**
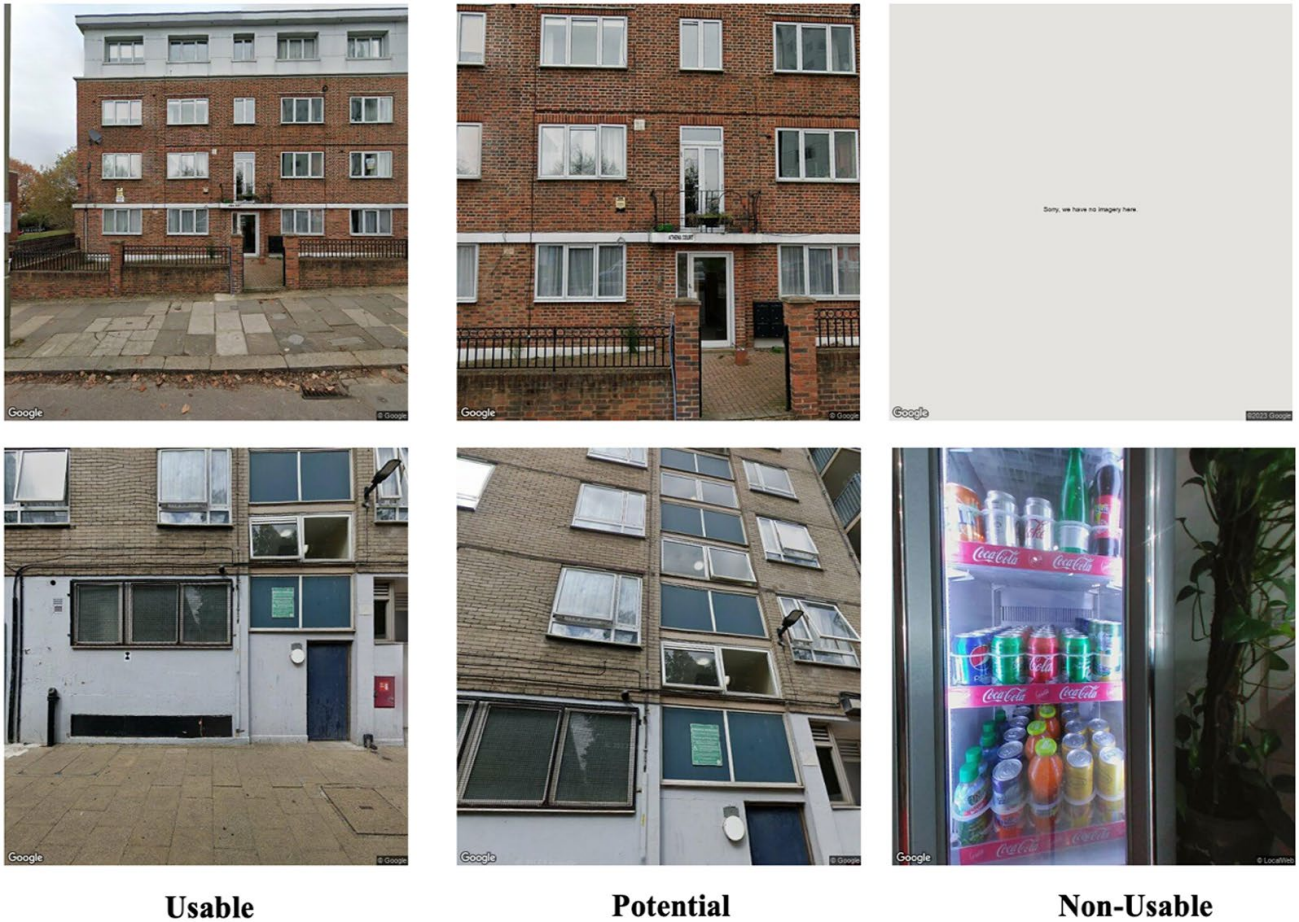
Examples of original dataset used in both tasks.

While the ranges for pitch and heading are consistent across tasks, FOV differs; images capturing the entire building façade require a wider zoom compared to those that focus only on the first story. The specific ranges for each parameter, applied in this study, are detailed in Table 2. The images were obtained at an output size of 640 × 640 pixels, which is the maximum resolution available on the GSV platform. In Fig. 5, the top row displays examples of images that are categorised as usable, potential, and non-usable, all of which provide a view of the entire building facade. Conversely, the bottom row presents images that show only the first story of the building, classified under the same categories.

**Table 2:** The camera parameters used in both tasks.

Parameters	Task1: whole building façade		Task2: the first story	
	Minimum	Maximum	Minimum	Maximum
FOV	10	35	30	55
Pitch	20	50	20	50
Heading	30	60	30	60

Although 1,000 distinct building addresses were initially selected, multiple images per address were retrieved by systematically adjusting critical camera parameters. In this research, the same address, acquired using different camera parameters, into both training, validation, and testing subsets are incorporated. This methodological choice explicitly aims to evaluate our model’s ability to identify and recommend suitable camera settings for the practical acquisition of building façade images, thus accurately reflecting real-world application scenarios.

Modifying these parameters created multiple unique perspectives for each building façade, thereby increasing the dataset size beyond the original number of addresses. Specifically, applying the camera parameter ranges resulted in 2,138 images for the whole-building façade task and 2,290 images for the first story building task.

The dataset was then divided into three subsets: a training set for model development, a validation set for selecting the optimal trained model, and a test set for evaluating the finalized model’s performance on unseen data. Images were randomly allocated into training (60%), validation (20%), and test (20%) subsets, resulting in 2,138, 712, and 712 images for the whole-building façade task, and 2,290, 763, and 763 images for the first-story building task, respectively. This random distribution ensured representativeness across all subsets.

#### Augmenting the dataset using training image augmentation methods

**Fig. 6:**
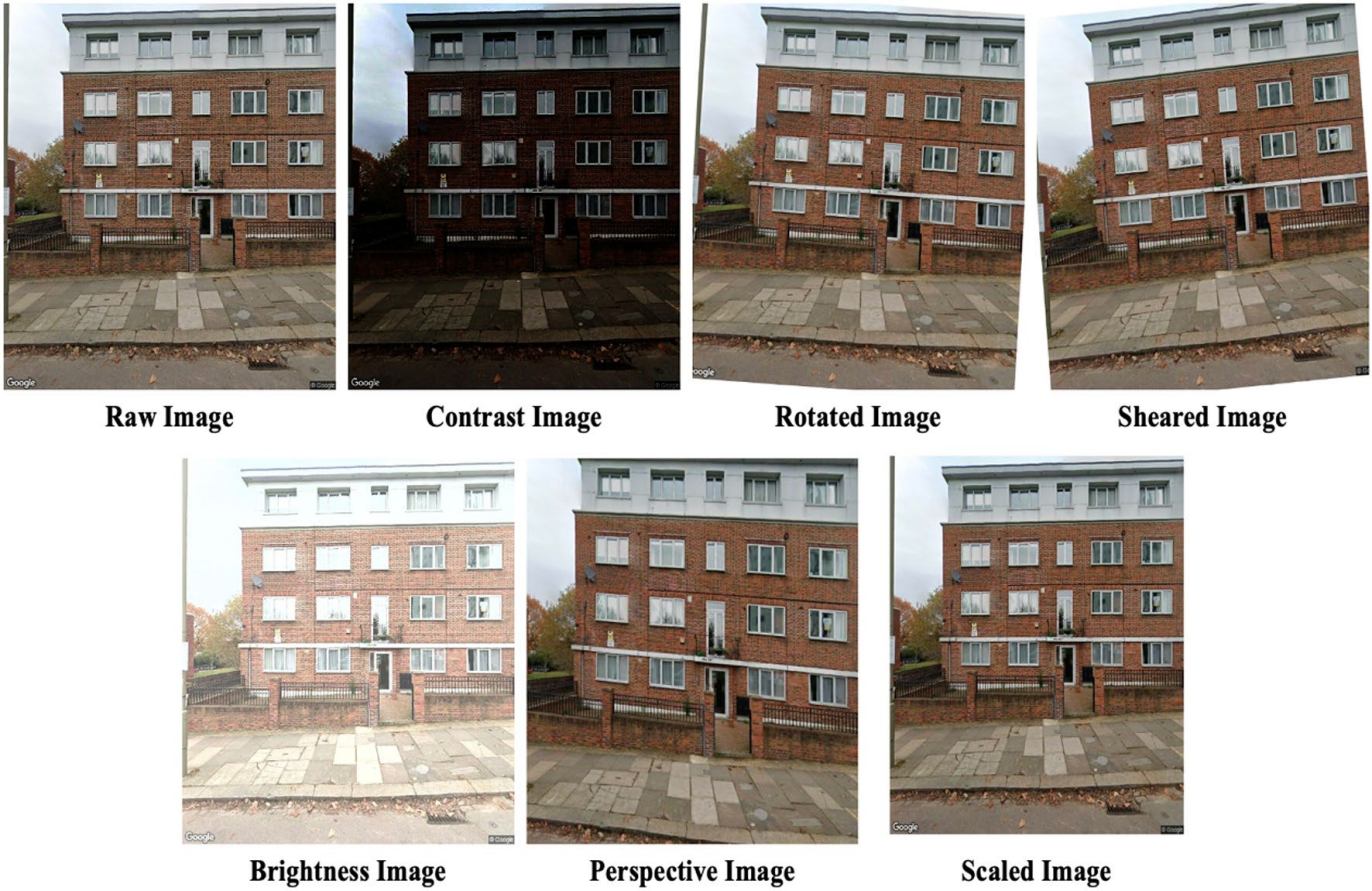
Examples of augmented images in a dataset of whole building façade.

**Fig. 7:**
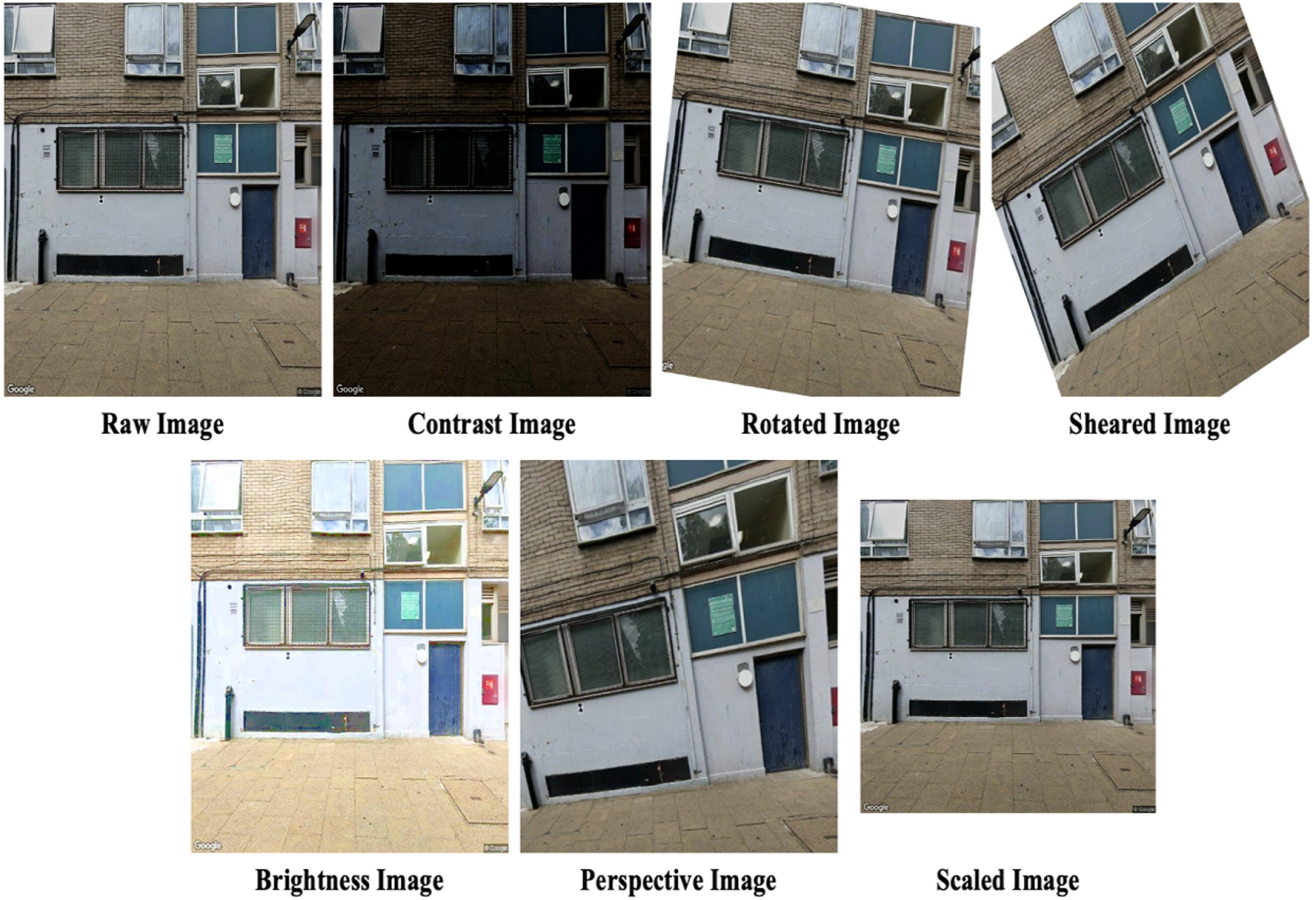
Examples of augmented images in a dataset of the first story.

The augmentation parameters were carefully selected through a trial-and-error approach to generate images closely resembling real-world conditions. As summarized in Table 3, the augmented images were created exclusively from the training datasets for each respective task. Figures 6 and 7 illustrate examples of the original and the corresponding augmented images for each task.

**Table 3:** Datasets created with ranges of parameters.

Dataset	Ranges of parameter	Number of images in whole building façade	Number of images in the first story
Brightness	[−30, 30]	2,138	2,290
Contrast	[0.5, 2.0]	2,138	2,290
Scale	[x: 0.8, 1.2], [y: 0.8, 1.2]	2,138	2,290
Perspective	[0.01, 0.15]	2,138	2,290
Rotation	[− 25°, 25°]	2,138	2,290
Shearing	[− 15°, 15°]	2,138	2,290
Total	-	12,828	13,740

#### Annotation

In image classification tasks, the annotation process involves assigning specific classes to ground truth data. In this research, annotation was carried out by analysing visual cues to determine whether the entire building façade or just the first story was observable in the images. Owing to the unique requirements of each task, the datasets were annotated individually. For both tasks, images were sorted into designated folders based on their classification as usable images, potential images, or non-usable images. To ensure the quality of the annotations, a separate team conducted a cross-check of a sample from the annotated images.

#### Synthesis of final dataset

To demonstrate the effectiveness of the proposed methods, two different datasets were constructed, and each dataset was divided into three data subsets: training data, validation data, and test data. Next, each image was annotated for the corresponding categories: usable images, potential images, and non-usable images. Table 4 shows the detailed distribution of annotated information for each dataset, respectively.

**Table 4:** Detailed distribution of datasets.

Dataset	Purpose	Number of images	Class		
			Usable	Potential	Non-usable
Whole building facade	Training	2,138	642	1,069	427
	Augmentation	12,828	3,852	6,414	2,562
	Validation	712	214	356	142
	Test	712	213	357	142
	Total	3,562	1,069	1,782	711
First story	Training	2,290	688	1,145	457
	Augmentation	13,740	4,128	6,870	2,742
	Validation	763	229	382	152
	Test	763	228	382	153
	Total	3,816	1,145	1,909	762

### Experimental settings

All experiments were mainly conducted on a system running Windows 10, equipped with an Intel Core i7-7700HQ processor (2.80 GHz, 8 threads), an NVIDIA GeForce GTX 3080 Ti GPU, and 32 GB of RAM. Python, along with the TensorFlow and Keras frameworks, was employed to implement and execute the deep learning algorithms. The dataset, along with detailed statistical information and the relevant code, including hyperparameters, is publicly accessible via the Figshare repository^2^.

## Results and discussion

### Results of training and validation

#### Assessment of underfitting, and overfitting

**Fig. 8:**
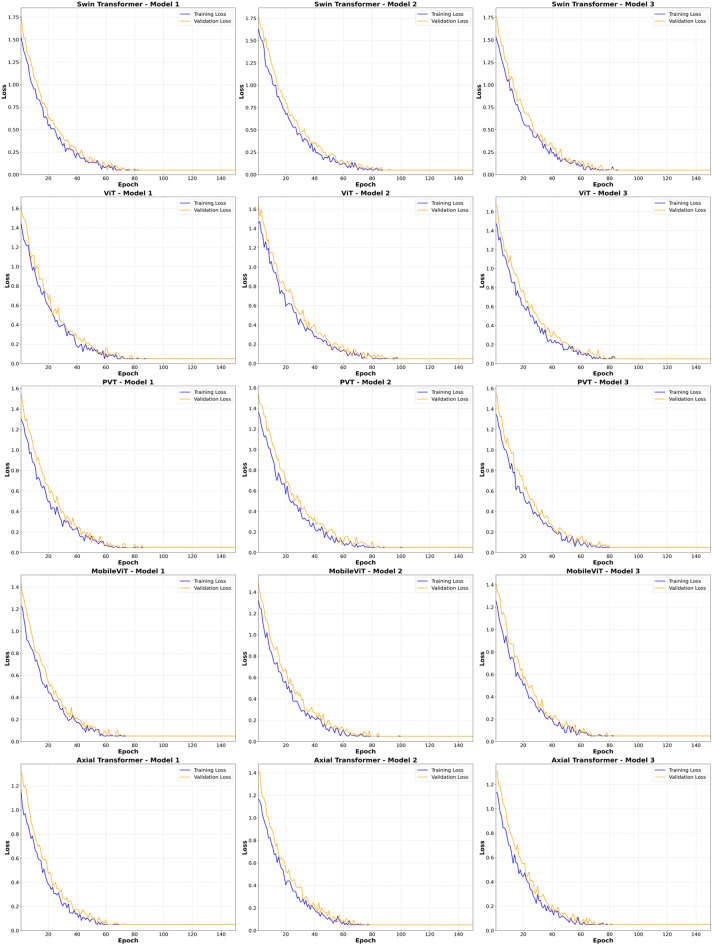
Examples of loss curves during training and validation for attention-based methods.

In this research, the analysis focused on the training and validation loss curves to identify possible underfitting or overfitting issues occurring throughout model training. Figure 8 illustrates representative examples of these curves for each transformer-based model at the point of maximum training epochs, clearly demonstrating their evolution during training.

All the models exhibited a consistent and gradual reduction in training and validation losses throughout the entire training duration, reflecting stable and effective learning processes. The continuous decrease observed in training losses confirmed that underfitting was not a significant concern. Furthermore, the similarity and concurrent trends observed in both training and validation losses implied that any overfitting was minimal or insignificant.

#### Overall performance

**Fig. 9:**
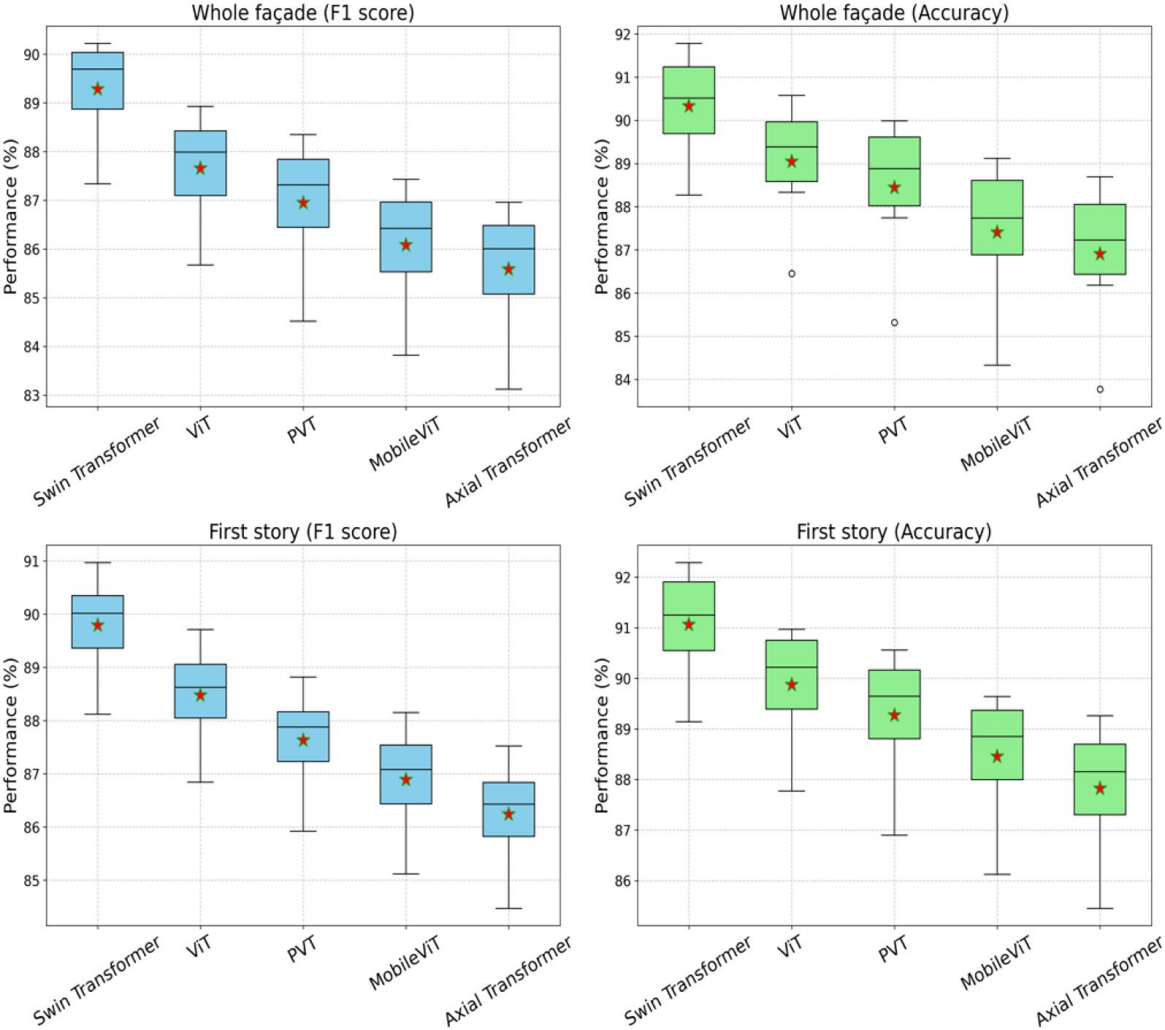
Boxplot of F1 score, and accuracy across methods by each hyperparameter.

The performance of five transformer-based methods—Swin Transformer, ViT, PVT, MobileViT, and Axial Transformer—was systematically evaluated for the classification of whole building façades and first-story images. The statistical distribution of these performance metrics (F1 score and accuracy) is visualized in the boxplots presented in Fig. 9, while detailed statistical measures are summarized in Table 5.

Swin Transformer outperformed other models across both tasks and performance metrics, achieving higher median and mean values for F1 scores and accuracy. In the whole building façade task, Swin Transformer recorded the highest mean F1 score (89.66%) and accuracy (90.49%). Conversely, Axial Transformer exhibited the lowest performance in this scenario, with a mean F1 score of 85.97% and mean accuracy of 87.19%, highlighting considerable variability among the evaluated transformer architectures.

For the first-story classification task, Swin Transformer again demonstrated superior performance, with the highest mean accuracy (91.23%) and a strong F1 score (90.29%). Axial Transformer, however, displayed the lowest mean accuracy (88.13%) and mean F1 score (86.74%).

The boxplots further illustrate minimal variation within models, indicating stable performance across multiple hyperparameter configurations. Swin Transformer’s narrower interquartile range and higher median values suggest robust and reliable classification capability, emphasizing its effectiveness and generalizability in comparison to the other evaluated transformer-based methods.

**Table 5:** Statistics of model performance based on F1 score and accuracy.

Task (Metric)	Model	Min	25%	Median	Mean	75%	Max	Std
Whole building façade (F1 score)	Swin Transformer	87.34	88.61	89.73	89.66	90.14	90.22	0.92
	ViT	85.67	86.81	88.03	87.95	88.56	88.93	1.01
	PVT	84.52	86.17	87.36	87.28	88	88.35	1.1
	MobileViT	83.82	85.25	86.46	86.39	87.13	87.43	1.12
	Axial Transformer	83.12	84.78	86.04	85.97	86.63	86.96	1.15
Whole building façade (Accuracy)	Swin Transformer	88.27	89.43	90.54	90.49	91.47	91.78	1.17
	ViT	86.45	88.33	89.43	89.34	90.14	90.58	1.38
	PVT	85.32	87.74	88.9	88.86	89.85	89.99	1.51
	MobileViT	84.33	86.61	87.75	87.72	88.9	89.12	1.54
	Axial Transformer	83.77	86.18	87.26	87.19	88.32	88.69	1.49
First story (F1 score)	Swin Transformer	88.12	89.24	90.37	90.29	90.97	89.74	1.08
	ViT	86.84	87.97	89.09	88.96	89.71	88.29	1.12
	PVT	85.92	87.08	88.2	88.07	88.82	87.69	1.18
	MobileViT	85.12	86.33	87.58	87.42	88.15	86.74	1.2
	Axial Transformer	84.47	85.72	86.87	86.74	87.52	86.12	1.22
First story (accuracy)	Swin Transformer	89.14	90.32	91.27	91.23	92.12	92.29	1.08
	ViT	87.77	89.13	90.25	90.19	90.97	90.92	1.12
	PVT	86.9	88.53	89.67	89.62	90.33	90.56	1.18
	MobileViT	86.12	87.72	88.88	88.82	89.53	89.64	1.2
	Axial Transformer	85.45	87.03	88.18	88.13	88.87	89.26	1.22

#### Analysis of class-wise performance

Table 6 presents class-wise performance results of the best-performing models for each transformer-based method evaluated across two classification tasks: whole-building façade classification and first-story façade classification. The results include F1 scores and accuracy for three distinct classes—Usable, Potential, and Non-usable—as well as their overall averages.

In the whole-building façade classification task, Swin Transformer demonstrated the highest class-wise performance, achieving F1 scores of 91.08% (Usable), 90.47% (Potential), and 89.12% (Non-usable), resulting in an overall average F1 score of 90.22%. Accuracy scores followed a similar trend, with the highest accuracy of 92.85% recorded for the Usable class, indicating robust performance across all classes. Conversely, Axial Transformer yielded the lowest performance among the evaluated methods, particularly for the Non-usable class, where it achieved an F1 score of 85.68% and accuracy of 87.42%.

For the first-story façade classification task, Swin Transformer again outperformed the other methods, achieving notably high accuracy of 93.87% for the Usable class and an overall average accuracy of 92.29%. Its class-wise F1 scores remained consistently high, demonstrating balanced and reliable classification performance. In contrast, MobileViT and Axial Transformer recorded comparatively lower class-wise performance. Axial Transformer, in particular, achieved the lowest F1 score (84.87%) and accuracy (87.37%) for the Non-usable class, indicating challenges in accurately classifying these images.

Overall, Swin Transformer exhibited superior and balanced performance across all evaluated classes. Models such as Axial Transformer and MobileViT showed noticeable performance variability and reduced effectiveness, particularly for challenging classes. These findings highlight Swin Transformer’s robust generalization capabilities and suitability for reliable use in both classification scenarios.

**Table 6:** Class-wise performance of best models in each method.

Task	Method	Metrics	Class			
			Usable	Potential	Non-usable	Average
Whole building façade	Swin Transformer	F1 score	91.08	90.47	89.12	90.22
		Accuracy	92.85	91.92	90.57	91.78
	ViT	F1 score	89.82	89.03	87.93	88.93
		Accuracy	91.67	90.76	89.32	90.58
	PVT	F1 score	89.27	88.52	87.25	88.35
		Accuracy	91.02	90.22	88.73	89.99
	MobileViT	F1 score	88.48	87.73	86.07	87.43
		Accuracy	90.22	89.27	87.88	89.12
	Axial Transformer	F1 score	87.98	87.22	85.68	86.96
		Accuracy	89.78	88.88	87.42	88.69
First story	Swin Transformer	F1 score	90.67	90.02	88.53	89.74
		Accuracy	93.87	92.48	90.52	92.29
	ViT	F1 score	89.27	88.53	87.08	88.29
		Accuracy	92.53	91.12	89.12	90.92
	PVT	F1 score	88.72	87.88	86.47	87.69
		Accuracy	92.03	90.72	88.92	90.56
	MobileViT	F1 score	87.83	86.97	85.43	86.74
		Accuracy	91.23	89.82	87.87	89.64
	Axial Transformer	F1 score	87.18	86.32	84.87	86.12
		Accuracy	90.83	89.57	87.37	89.26

#### Selection of best method

Table 7 summarizes the 5-fold cross-validation performance results of the best-performing transformer-based models (Swin Transformer, ViT, PVT, MobileViT, and Axial Transformer) for two key classification tasks: whole-building façade classification and first-story façade classification. Swin Transformer achieved the highest average performance across both tasks, demonstrating robust generalization capability with minimal variability among folds. Specifically, Swin Transformer recorded the highest average F1 scores (90.15% for whole-building façades and 89.66% for first-story façades) and the highest average accuracy scores (91.69% and 92.21%, respectively).

Conversely, Axial Transformer exhibited the lowest overall performance, with average F1 scores of 86.87% for whole-building façades and 86.03% for first-story façades, indicating comparatively limited effectiveness. Performance variations across folds were modest, signifying consistent and reliable stability.

Regarding computational efficiency, Tables S.2 (GPU inference times) and S.3 (CPU inference times) summarize the detection speeds for each transformer-based method. Swin Transformer consistently demonstrated the fastest detection speeds, averaging 0.0221 s per instance on GPU and 0.3315 s per instance on CPU. ViT and Axial Transformer showed slightly slower performances, with mean inference times of 0.0235 and 0.0239 s per instance on GPU, and 0.3525 and 0.3585 s per instance on CPU, respectively. MobileViT had moderate detection speeds, averaging 0.0248 s on GPU and 0.3720 s on CPU. The slowest inference times were observed for PVT, averaging 0.0272 s per instance on GPU and 0.4080 s per instance on CPU.

Considering predictive accuracy and computational efficiency together, Swin Transformer emerges as the most suitable model for practical deployment in façade and first-story image classification tasks. Its superior combination of high accuracy, consistency, and rapid inference speed highlights its strong potential for reliable real-world applications.

**Table 7:** 5-fold cross-validation results of best-performing models in each method.

Task	Metric	Method	Fold 1	Fold 2	Fold 3	Fold 4	Fold 5	Mean
Whole building façade	F1 score	Swin Transformer	90.22	90.09	90.22	90.22	90	90.15
		ViT	88.93	88.79	88.93	88.93	88.69	88.85
		PVT	88.35	88.2	88.35	88.35	88.09	88.27
		MobileViT	87.43	87.28	87.43	87.43	87.17	87.35
		Axial Transformer	86.96	86.8	86.96	86.96	86.69	86.87
	Accuracy	Swin Transformer	91.78	91.62	91.78	91.78	91.51	91.69
		ViT	90.58	90.39	90.58	90.58	90.26	90.48
		PVT	89.99	89.78	89.99	89.99	89.64	89.88
		MobileViT	89.12	88.91	89.12	89.12	88.76	89.01
		Axial Transformer	88.69	88.48	88.69	88.69	88.34	88.58
First story	F1 score	Swin Transformer	89.74	89.59	89.74	89.74	89.49	89.66
		ViT	88.29	88.14	88.29	88.29	88.03	88.21
		PVT	87.69	87.53	87.69	87.69	87.41	87.6
		MobileViT	86.74	86.57	86.74	86.74	86.46	86.65
		Axial Transformer	86.12	85.95	86.12	86.12	85.83	86.03
	Accuracy	Swin Transformer	92.29	92.14	92.29	92.29	92.04	92.21
		ViT	90.92	90.77	90.92	90.92	90.66	90.84
		PVT	90.56	90.4	90.56	90.56	90.28	90.47
		MobileViT	89.64	89.47	89.64	89.64	89.36	89.55
		Axial Transformer	89.26	89.09	89.26	89.26	88.97	89.17

### Performance analysis of best-performing model

#### Validation and test dataset analysis

**Fig. 10:**
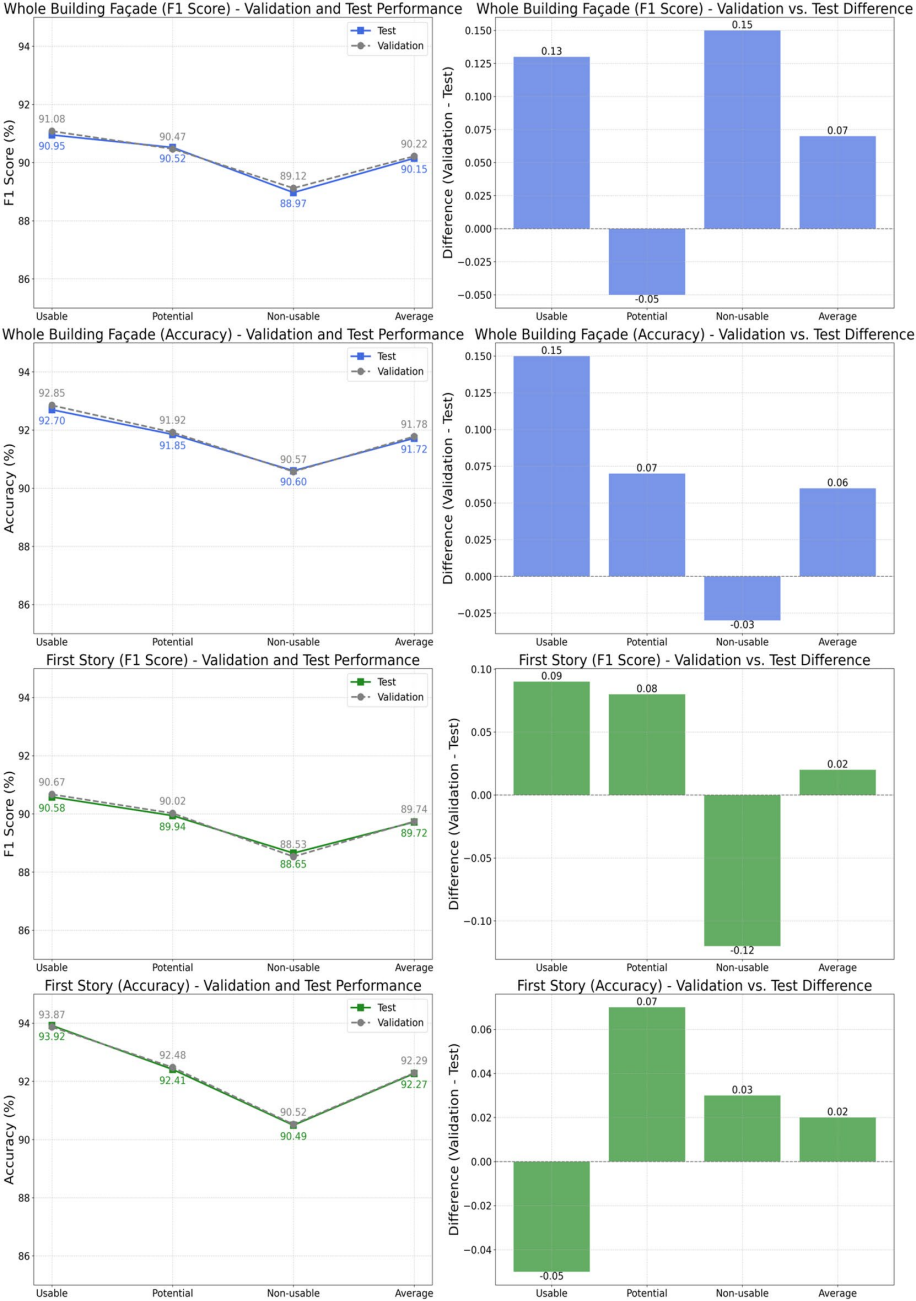
Performance variations of the best-performing model between validation and test sets.

Figure 10 represents the results of comparison about the Swin Transformer’s performance between validation and test datasets for two tasks: whole building façade and first story classifications. In the whole building façade task, minor variations appeared, with validation and test averages closely matching (F1: 90.22% vs. 90.15%; accuracy: 91.78% vs. 91.72%), indicating stable performance. Similarly, the first-story task displayed negligible differences (F1: 89.74% vs. 89.72%; accuracy: 92.29% vs. 92.27%). Class-wise analysis also revealed minimal fluctuations, with minor increases and decreases in specific classes, suggesting strong generalization rather than overfitting. The minimal differences (within ± 0.15%) between datasets highlight the model’s robustness and consistent predictive ability across diverse, unseen data, confirming its suitability for practical applications.

#### Impact of image conditions

Table 8 presents model performance across varying image conditions, highlighting the impact of complexity factors such as illumination, visual range, architectural style, building density, and occlusion. Performance was higher under favorable conditions. In both tasks, the model demonstrated reduced effectiveness under low illumination compared to high illumination, with F1 scores decreasing notably from 91.23 to 90.25 (whole façade) and 90.72 to 89.80 (first story). Similarly, accuracy dropped from 92.74 to 91.82% and from 93.56 to 92.78%, respectively. Near-field views significantly outperformed far-field views, indicating a clear challenge with distant images.

Moderate differences appeared concerning architectural styles and building densities, with traditional buildings and single-density scenarios yielding superior results. Occlusions by cars or other obstacles markedly impacted performance, reducing accuracy in the whole façade task from 93.11% (no occlusion) to 91.45% (occluded), and from 93.89 to 92.45% in the first-story scenario. Based on the resulting findings, the model exhibited robust generalization but was sensitive to challenging conditions involving low illumination, greater distances, and partial occlusions.

**Table 8:** Results of the best model, grouped by different image conditions.

Task	Image conditions	Ranges	Class					
			F1 score			Accuracy		
			Usable	Potential	Average	Usable	Potential	Average
Whole building facade	Illumination	High	91.37	91.08	91.23	93.16	92.31	92.74
		Low	90.53	89.96	90.25	92.24	91.39	91.82
	Visual range	Near-field view	91.64	91.25	91.45	93.41	92.56	92.99
		Far-field view	90.26	89.79	90.03	91.99	91.14	91.57
	Architectural style	Traditional	91.22	90.91	91.07	93.02	92.08	92.55
		Modern	90.68	90.13	90.41	92.38	91.62	92.00
	Density of building	Single	91.49	91.11	91.30	93.27	92.41	92.84
		Multiple	90.41	89.93	90.17	92.13	91.29	91.71
	Occlusion	No occlusion	91.71	91.33	91.52	93.53	92.68	93.11
		Occluded	90.19	89.71	89.95	91.87	91.02	91.45
First story	Illumination	High	91.02	90.42	90.72	94.22	92.89	93.56
		Low	90.14	89.46	89.80	93.62	91.93	92.78
	Visual range	Near-field view	91.21	90.61	90.91	94.41	93.15	93.78
		Far-field view	89.95	89.27	89.61	93.43	91.67	92.55
	Architectural style	Traditional	90.85	90.25	90.55	94.03	92.72	93.38
		Modern	90.31	89.63	89.97	93.81	92.1	92.96
	Density of building	Single	91.07	90.49	90.78	94.29	93.05	93.67
		Multiple	90.09	89.39	89.74	93.55	91.77	92.66
	Occlusion	No occlusion	91.32	90.76	91.04	94.51	93.26	93.89
		Occluded	89.84	89.12	89.48	93.33	91.56	92.45

#### Application for street view maps

**Fig. 11:**
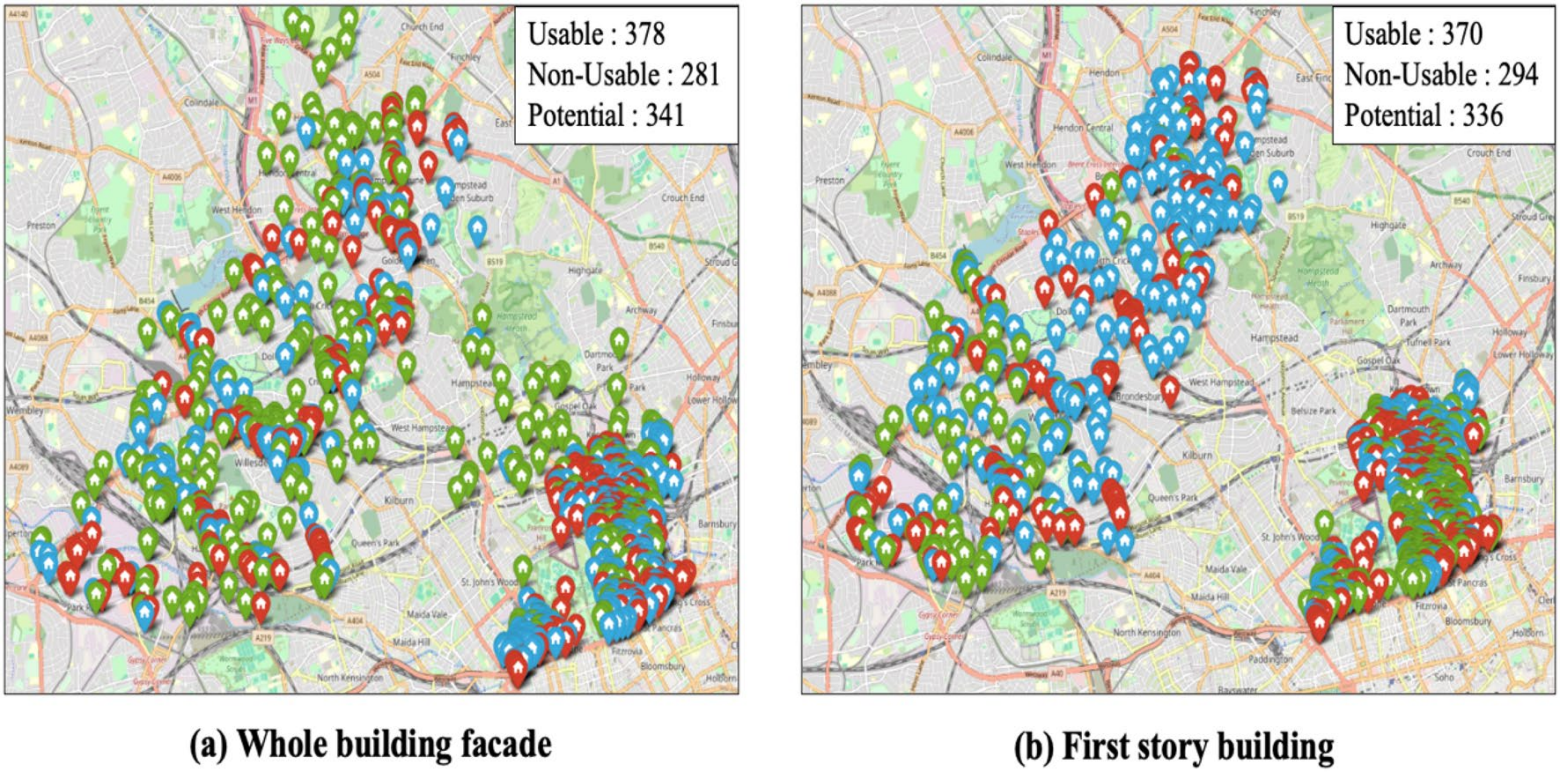
Application examples of best-performing model (Map data © 2025 Google.).

The trained models for each task—Task 1 (whole-building façade images) and Task 2 (first-story façade images)—were applied to the complete experimental dataset, comprising 1,000 images per task. A total of 10 iterations were conducted to adjust camera parameters. Detection results were geocoded, and each class (usable, potential, and non-usable) was visualized on Google Maps using color-coded markers: green for usable, blue for potential, and red for non-usable, as illustrated in Fig. 11a and b.

For Task 1, the discrepancies between ground truth labels and model predictions were as follows:

- Ground Truth: Usable: 392, Potential: 328, Non-usable: 280.
- Model Predictions: Usable: 378, Potential: 355, Non-usable: 267.

For Task 2, the discrepancies were:

- Ground Truth: Usable: 377, Potential: 332, Non-usable: 291.
- Model Predictions: Usable: 352, Potential: 336, Non-usable: 312.

#### Grad-cam analysis

**Fig. 12:**
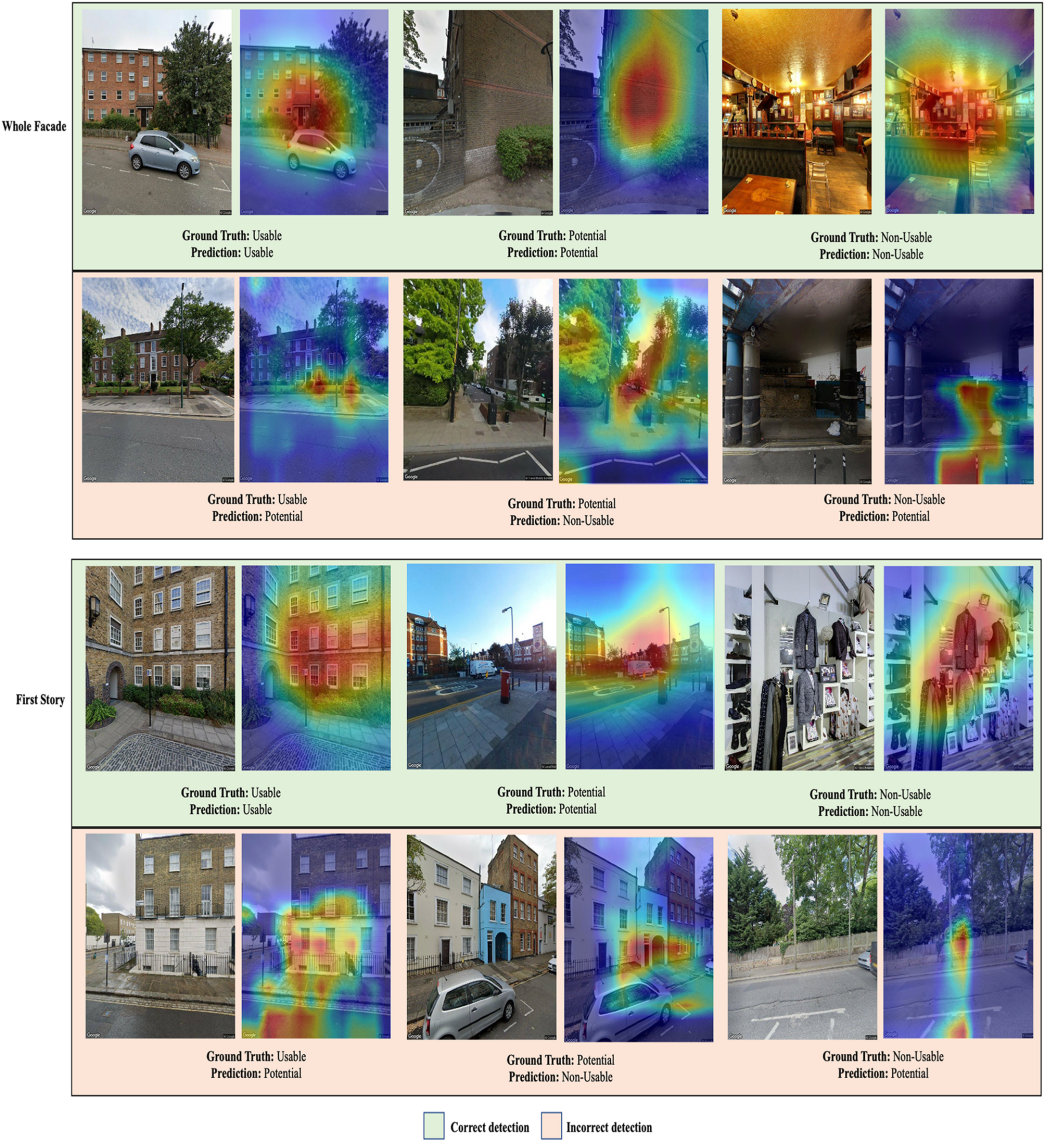
Examples of Grad-CAM analysis for correctly and incorrectly detected results.

To further examine these findings, a Gradient-weighted Class Activation Mapping (Grad-CAM) analysis was carried out. Figure 12 shows illustrative Grad-CAM heatmaps obtained from the top-performing Swin Transformer-based model.

For correctly classified images in both tasks, the “usable” class prominently exhibited focused activations primarily on building façades. In contrast, the “potential” class displayed activations that were generally directed towards façade regions but were often scattered or inconsistently aligned. The “non-usable” class consistently showed strong activations targeting irrelevant or obstructive features, such as trees, and vehicles.

For incorrectly classified images, activations in the “usable” class appeared dispersed across façade areas, likely due to factors such as uncommon architectural designs, lighting variations, or reflections. In the “potential” class, activation patterns were unclear or variable, emphasizing the model’s challenges with ambiguous cases. Lastly, in the “non-usable” class, there was a noticeable lack of emphasis on obstructions or distracting features, reflecting misinterpretations by the model.

### Comparative analysis

#### Selection of comparative methods

To also illustrate the effectiveness of the proposed Switn Transformert-based approach, comparative analyses were performed using various established classification algorithms based on non-attention-based method, including ResNet-101, ResNet-152, MobileNetV3, CSPNet, ConvNeXt, and EfficientNet. Hyperparameters for each of these algorithms were carefully tuned through a series of preliminary experiments, ultimately leading to the evaluation of 198 different model configurations. Table S.1 provides a concise overview of the classification methods used, their selected optimal hyperparameters, and the best-performing model configurations as determined from testing results.

#### Comparative results

##### F1score, and accuracy with detection speed

Table 9 presents a comparative evaluation of the proposed method and several popular classifiers across two image-classification tasks: whole building façade and first-story analysis. The proposed transformer-based model achieved superior performance compared to alternative architectures such as ResNet-101, ResNet-152, MobileNetv3, CSPNet, ConvNeXt, and EfficientNet.

Specifically, for the whole building façade task, the proposed method attained the highest average F1 score (90.15%) and accuracy (91.72%). This surpasses the second-best model, ConvNeXt, by approximately 0.5% points in both F1 score and accuracy. In the first-story task, the proposed method again delivered the best results, with an average F1 score of 89.72% and accuracy of 92.27%. It notably outperformed the second-best architecture, ConvNeXt, by approximately 0.44% points in F1 score and 0.33% points in accuracy.

As shown in Tables S.2 and S.3, the proposed transformer-based model achieved the highest accuracy and F1 scores, outperforming the second-best model, ConvNeXt, by approximately 0.5% points in the whole building façade task and around 0.4% points in the first-story task. Although the proposed method exhibited slightly slower detection speeds (GPU: 0.0221 s/instance; CPU: 0.3315 s/instance) compared to architectures such as MobileNetv3 and ResNet-101, these differences were minimal.

**Table 9:** Comparative results of proposed method and other classifiers.

Task	Architecture	Usable		Potential		Non-usable		Average	
		F1 score	Accuracy	F1 score	Accuracy	F1 score	Accuracy	F1 score	Accuracy
Whole building façade	Proposed method	90.95	92.7	90.52	91.85	88.97	90.6	90.15	91.72
	ResNet-101	89.72	91.25	89.28	90.63	87.52	89.17	88.84	90.35
	ResNet-152	90.02	91.68	89.61	90.92	88.1	89.53	89.24	90.71
	MobileNetv3	89.25	90.83	88.74	90.07	86.83	88.43	88.27	89.78
	CSPNet	89.83	91.46	89.4	90.78	87.85	89.3	89.03	90.51
	ConvNeXt	90.45	92.19	90.06	91.44	88.43	90.02	89.65	91.22
	EfficientNet	89.56	91.05	89.12	90.42	87.62	89.05	88.77	90.17
First story	Proposed method	90.58	93.92	89.94	92.41	88.65	90.49	89.72	92.27
	ResNet-101	89.33	92.5	88.62	91.04	87.47	89.11	88.47	90.88
	ResNet-152	89.78	93.05	89.1	91.69	88	89.76	88.96	91.5
	MobileNetv3	88.91	92.17	88.2	90.7	86.95	88.65	88.02	90.51
	CSPNet	89.46	92.81	88.75	91.33	87.72	89.41	88.64	91.18
	ConvNeXt	90.1	93.67	89.47	92.13	88.27	90.03	89.28	91.94
	EfficientNet	89.12	92.33	88.5	90.91	87.25	88.95	88.29	90.73

##### Bootstrap analysis

A paired bootstrap analysis was conducted to verify whether small performance differences (≤ 1% point) between models were statistically significant. Table 10 summarizes the results of this analysis. For the whole-building façade classification task, statistically significant differences were observed between the best-performing model and MobileNetV3 (95% CI: [0.0070, 0.0660]) as well as EfficientNet (95% CI: [0.0028, 0.0618]). Conversely, comparisons with ResNet-101, ResNet-152, CSPNet, and ConvNeXt did not show statistically significant differences, as their 95% confidence intervals included zero. For the first-story façade task, none of the model comparisons revealed statistically significant differences, with all corresponding confidence intervals encompassing zero. Overall, these findings indicate that, despite minor numerical differences (≤ 1% point) between models, most were not statistically significant. This emphasizes the importance of statistical validation rather than solely relying on numerical differences.

**Table 10:** Paired bootstrap analysis results.

Task	Model comparison with best-performing model	95% CI lower	95% CI upper	Significance
Whole building façade	ResNet-101	−0.0014045	0.05758427	False
	ResNet-152	0	0.05898876	False
	MobileNetv3	0.00702247	0.06601124	True
	CSPNet	0	0.05898876	False
	ConvNeXt	−0.0042135	0.05196629	False
	EfficientNet	0.00280899	0.06179775	True
First story	ResNet-101	−0.0262123	0.03145478	False
	ResNet-152	−0.0314548	0.02359109	False
	MobileNetv3	−0.0262123	0.03014417	False
	CSPNet	−0.0301442	0.0249017	False
	ConvNeXt	−0.0406291	0.01441678	False
	EfficientNet	−0.0262123	0.02883355	False

## Conclusions

This study developed transformer-based deep learning models designed for automatically classifying SSVIs into three categories—usable, potential, and non-usable—for building characteristic analyses. Specifically, two critical tasks were targeted: the analysis of entire building façades and detailed inspections of first-story façades.

Five transformer architectures (Swin Transformer, ViT, PVT, MobileViT, and Axial Transformer) were combined with extensive hyperparameter tuning and six data augmentation techniques, yielding a total of 1,026 models. The main finding is that, among these, the proposed transformer-based approach demonstrated the highest overall performance. Specifically, for the whole building façade task, the proposed model achieved class-wise F1 scores of 90.95% (usable), 90.52% (potential), and 88.97% (non-usable), with corresponding accuracies of 92.70%, 91.85%, and 90.60%, respectively, resulting in an average F1 score of 90.15% and accuracy of 91.72%. For the first-story façade task, the model similarly exhibited superior performance, obtaining class-wise F1 scores of 90.58% (usable), 89.94% (potential), and 88.65% (non-usable), along with accuracies of 93.92%, 92.41%, and 90.49%, respectively, yielding an average F1 score of 89.72% and accuracy of 92.27%.

Comparative analysis showed that transformer-based models consistently outperformed 810 traditional CNN-based architectures (including ResNet, MobileNet, CSPNet, ConvNeXt, and EfficientNet) in both accuracy and F1 score. Moreover, the best-performing transformer model demonstrated rapid detection capabilities, with an average inference time of 0.022 s per image, underscoring its practical suitability for real-time analysis. Finally, paired bootstrap analysis indicated statistically significant performance differences between the proposed transformer-based model and MobileNetv3 and EfficientNet for the whole building façade task; however, no significant differences were observed for the first-story façade task, as all corresponding confidence intervals included zero.

This study highlights the significant practical benefits of the proposed transformer-based solution, including enhanced efficiency and accuracy in classifying Static Street View Images (SSVIs) for urban building analysis. Additionally, future research should prioritize integrating this method with Geographic Information System (GIS) platforms. Such integration could facilitate comprehensive spatial analyses, enhance real-time urban planning capabilities, and support scalability across diverse geographic contexts.

Although promising, the developed models are limited by the dataset’s geographical specificity and temporal scope, potentially affecting their generalizability. Furthermore, this research employed only traditional image augmentation techniques, highlighting the need to investigate advanced augmentation methods—such as GAN-based augmentation or sophisticated geometric transformations—in future studies. Therefore, subsequent research should focus on expanding dataset diversity, employing advanced augmentation strategies, and leveraging robust pre-trained transformer models to enable more efficient and broadly applicable façade analyses.

## Supplementary Information

Below is the link to the electronic supplementary material.

Supplementary Material 1

## Data Availability

The datasets generated during and/or analysed during the current study are available from the corresponding author on reasonable request.

## References

[1] Gong, F. Y. et al. Mapping sky, tree, and Building view factors of street canyons in a high-density urban environment. *Build. Environ.*10.1016/j.buildenv.2018.02.042 (2018).

[2] Wang, S., Park, S., Park, S. & Kim, J. Building façade datasets for analyzing Building characteristics using deep learning. *Data Br.* **57**, 110885 (2024). 10.1016/j.dib.2024.110885 PMC11415630 39309718

[3] Zou, S. & Wang, L. Detecting individual abandoned houses from Google street view: A hierarchical deep learning approach. *ISPRS J. Photogramm Remote Sens.*10.1016/j.isprsjprs.2021.03.020 (2021).

[4] Ghasemian Sorboni, N., Wang, J. & Najafi, M. R. Fusion of Google street view, lidar, and orthophoto classifications using ranking classes based on F1 score for Building Land-Use type detection. *Remote Sens.* **16** (2024).

[5] Wu, M., Huang, Q., Gao, S. & Zhang, Z. Mixed land use measurement and mapping with street view images and Spatial context-aware prompts via zero-shot multimodal learning. *Int. J. Appl. Earth Obs Geoinf.*10.1016/j.jag.2023.103591 (2023).

[6] Wang, S. & Han, J. Automated detection of exterior cladding material in urban area from street view images using deep learning. *J. Build. Eng.* **96**, 110466 (2024).

[7] Chen, X., Ding, X. & Ye, Y. Mapping sense of place as a measurable urban identity: using street view images and machine learning to identify Building façade materials. *Environ. Plan. B Urban Anal. City Sci.* **52**, 965–984 (2025).

[8] Xu, F., Wong, M. S., Zhu, R., Heo, J. & Shi, G. Semantic segmentation of urban Building surface materials using multi-scale contextual attention network. *ISPRS J. Photogramm Remote Sens.*10.1016/j.isprsjprs.2023.06.001 (2023).

[9] Li, W. et al. Fine-grained Building function recognition with street-view images and GIS map data via geometry-aware semi-supervised learning. *Int. J. Appl. Earth Obs Geoinf.* **137**, 104386 (2025).

[10] Chen, F. C., Subedi, A., Jahanshahi, M. R., Johnson, D. R. & Delp, E. J. Deep Learning–Based Building attribute Estimation from Google street view images for flood risk assessment using feature fusion and task relation encoding. *J. Comput. Civ. Eng.*10.1061/(asce)cp.1943-5487.0001025 (2022).

[11] Kalfarisi, R., Hmosze, M. & Wu, Z. Y. Detecting and geolocating City-Scale Soft-Story buildings by deep machine learning for urban seismic resilience. *Nat. Hazards Rev.*10.1061/(asce)nh.1527-6996.0000541 (2022).

[12] Han, J., Kim, J., Kim, S. & Wang, S. Effectiveness of image augmentation techniques on detection of Building characteristics from street view images using deep learning. *J. Constr. Eng. Manag*. **150**, 1–18 (2024).

[13] Li, Y., Chen, Y., Rajabifard, A., Khoshelham, K. & Aleksandrov, M. Estimating building age from google street view images using deep learning. in Leibniz International Proceedings in Informatics, LIPIcs (2018). 10.4230/LIPIcs.GIScience.2018.40

[14] Sun, M., Zhang, F., Duarte, F. & Content, D. Automatic Building Age Prediction from Street View Images. in Proceedings of 2021 7th IEEE International Conference on Network Intelligence and IC-NIDC (2021). (2021) 10.1109/IC-NIDC54101.2021.9660554

[15] Benz, A., Voelker, C., Daubert, S. & Rodehorst, V. Towards an automated image-based Estimation of Building age as input for Building energy modeling (BEM). *Energy Build.*10.1016/j.enbuild.2023.113166 (2023).

[16] Wang, P. et al. An automatic Building façade deterioration detection system using infrared-visible image fusion and deep learning. *J. Build. Eng.* **95**, 110122 (2024).

[17] Chen, Y. et al. BFA-YOLO: A balanced multiscale object detection network for Building façade elements detection. *Adv. Eng. Inf.* **65**, 103289 (2025).

[18] Cao, M. T. Drone-assisted segmentation of tile peeling on Building façades using a deep learning model. *J. Build. Eng.*10.1016/j.jobe.2023.108063 (2023).

[19] Kim, J., Kamari, M., Lee, S. & Ham, Y. Large-Scale visual Data–Driven probabilistic risk assessment of utility Poles regarding the vulnerability of power distribution infrastructure systems. *J. Constr. Eng. Manag*. 10.1061/(asce)co.1943-7862.0002153 (2021).

[20] Hu, C. B., Zhang, F., Gong, F. Y., Ratti, C. & Li, X. Classification and mapping of urban Canyon geometry using Google street view images and deep multitask learning. *Build. Environ.*10.1016/j.buildenv.2019.106424 (2020).33132484

[21] Li, X. & Ratti, C. Using google street view for street-level urban form analysis, a case study in Cambridge, Massachusetts. in Modeling and Simulation in Science, Engineering and Technology (2019). 10.1007/978-3-030-12381-9_20

[22] Bai, Y., Cao, M., Wang, R., Liu, Y. & Wang, S. How street greenery facilitates active travel for university students. *J. Transp. Heal*. 10.1016/j.jth.2022.101393 (2022).

[23] Cha, Y. J. & Wang, Z. Unsupervised novelty detection–based structural damage localization using a density peaks-based fast clustering algorithm. *Struct. Heal Monit.*10.1177/1475921717691260 (2018).

[24] Wang, Z. & Cha, Y. J. Unsupervised deep learning approach using a deep auto-encoder with a one-class support vector machine to detect damage. *Struct. Heal Monit.*10.1177/1475921720934051 (2021).

[25] Wang, Z. & Cha, Y. Unsupervised machine and deep learning methods for structural damage detection: A comparative study. *Eng. Rep.*10.1002/eng2.12551 (2022).

[26] Eum, I., Kim, J., Wang, S. & Kim, J. Heavy equipment detection on construction sites using you only look once (YOLO-Version 10) with transformer architectures. *Appl. Sci.* **15** (2025).

[27] Park, S., Kim, J., Wang, S. & Kim, J. Effectiveness of image augmentation techniques on non-protective personal equipment detection using YOLOv8. *Appl. Sci.* **15** (2025).

[28] Hwang, D., Kim, J. J., Moon, S. & Wang, S. Image augmentation approaches for building dimension estimation in street view images using object detection and instance segmentation based on deep learning. *Appl. Sci.* **15** (2025).

[29] Wang, S., Korolija, I. & Rovas, D. Impact of traditional augmentation methods on window state detection. *CLIMA 2022 Conf.* **1-8**10.34641/clima.2022.375 (2022).

[30] Wang, S., Eum, I., Park, S. & Kim, J. A semi-labelled dataset for fault detection in air handling units from a large-scale office. *Data Br.* **57**, 110956 (2024). 10.1016/j.dib.2024.110956 PMC11460478 39381011

[31] Xiao, B. & Kang, S. C. Development of an Image Data Set of Construction Machines for Deep Learning Object Detection. *J. Comput. Civ. Eng.* doi:10.1061/(asce)cp.1943-5487.0000945. (2021).

[32] Wang, C. Y., Yeh, I. H. & Mark Liao, H. Y. Yolov9: Learning what you want to learn using programmable gradient information. in European Conference on Computer Vision 1–21Springer, (2025).

[33] Sharma, A., Kumar, V. & Longchamps, L. Comparative performance of YOLOv8, YOLOv9, YOLOv10, YOLOv11 and faster R-CNN models for detection of multiple weed species. *Smart Agric. Technol.* **9**, 100648 (2024).

[34] Alzubaidi, L. et al. Review of deep learning: concepts, CNN architectures, challenges, applications, future directions. *J. Big Data*. 10.1186/s40537-021-00444-8 (2021). 33816053 10.1186/s40537-021-00444-8 PMC8010506

[35] Wang, S. Automated fault diagnosis detection of air handling units using real operational labelled data and Transformer-based methods at 24-hour operation hospital. *Build. Environ.* **113257**10.1016/j.buildenv.2025.113257 (2025).

[36] Wang, S., Kim, J., Park, S. & Kim, J. Fault diagnosis of air handling units in an auditorium using real operational labeled data across different operation modes. *Comput. Civ. Eng.* **39** (2025).

[37] Krizhevsky, A., Sutskever, I. & Hinton, G. E. ImageNet classification with deep convolutional neural networks. *Adv. Neural Inf. Process. Syst.* 1–9. 10.1016/j.protcy.2014.09.007 (2012).

[38] Wang, S. Evaluation of impact of image augmentation techniques on two tasks: window detection and window States detection. *Results Eng.* **24**, 103571 (2024).

[39] Kandel, I., Castelli, M. & Manzoni, L. Brightness as an augmentation technique for image classification. *Emerg. Sci. J.*10.28991/ESJ-2022-06-04-015 (2022).

[40] Shorten, C. & Khoshgoftaar, T. M. A survey on Image Data Augmentation for Deep Learning. *J. Big Data* doi:10.1186/s40537-019-0197-0. (2019). 10.1186/s40537-021-00492-0 PMC8287113 34306963

[41] Wang, S., Eum, I., Park, S. & Kim, J. A labelled dataset for rebar counting inspection on construction sites using unmanned aerial vehicles. *Data Br.* **110720**10.1016/j.dib.2024.110720 (2024). 10.1016/j.dib.2024.110720 PMC11295459 39100779

[42] Ottoni, A. L. C., de Amorim, R. M., Novo, M. S. & Costa, D. B. Tuning of data augmentation hyperparameters in deep learning to Building construction image classification with small datasets. *Int. J. Mach. Learn. Cybern*. 10.1007/s13042-022-01555-1 (2023). 35432624 10.1007/s13042-022-01555-1 PMC9005628

[43] Wang, S., Hae, H. & Kim, J. Development of easily accessible electricity consumption model using open data and GA-SVR. *Energies*10.3390/en11020373 (2018).

[44] Wang, S., Kim, M., Hae, H., Cao, M. & Kim, J. The development of a Rebar-Counting model for reinforced concrete columns: using an unmanned aerial vehicle and Deep-Learning approach. *J. Constr. Eng. Manag*. **149**, 1–13 (2023).

[45] Wang, S., Moon, S., Eum, I., Hwang, D. & Kim, J. A text dataset of fire door defects for pre-delivery inspections of apartments during the construction stage. *Data Br.* **60**, 111536 (2025). 10.1016/j.dib.2025.111536 PMC12019833 40275975

