# Supplementary material for: Development of approach to an automated acquisition of static street view images using transformer architecture for analysis of Building characteristics
Source: Sci Rep. 2025 Aug 8;15:29062. doi: 10.1038/s41598-025-14786-3 (PMC12334604; doi:10.1038/s41598-025-14786-3)
Supplement: Supplementary file 1 — Supplementary Material 1 [file 41598_2025_14786_MOESM1_ESM.docx]

**Supplementary Tables**

**Table S.1** Detail of used hyperparameters in each method

| Method | Used hyperparameters | | Numbers of models | Best hyperparameter |
| --- | --- | --- | --- | --- |
| Swin Transformer | Epochs: [100,150], Batch size: [32,64,128], Learning rate: [0.0001,0.0005,0.001] | Window size: [7,12], Depth: [2,4,6], Embed dim: [96, 192] | 216 | Epochs:150, Batch:64, Learning rate:0.0005, Window size:7, Depth:6, Embed:128 |
| ViT | Epochs: [100,150], Batch size: [32,64,128], Learning rate: [0.0001,0.0005,0.001] | Patch size: [8, 16, 32], Transformer layers: [8,12], Hidden dim: [256,384] | 216 | Epochs:150, Batch size:64, Learning rate:0.0005, Patch size:16, Transformer layers:12, Hidden dim:384 |
| PVT | Epochs: [100,150], Batch size: [32,64,128], Learning rate: [0.0001,0.0005,0.001] | Pyramid stages: [2,3,4], Embed dim: [64,128,256] | 162 | Epochs:150, Batch size:64, LR:0.001, Pyramid stages:4, Embed dim:128 |
| MobileViT | Epochs: [100,150], Batch size: [32,64,128], Learning rate: [0.0001,0.0005,0.001] | MobileViT blocks: [2,4,6], Transformer blocks: [2,3], Embed dim: [64,128] | 216 | Epochs:150, Batch size:64, Learning rate:0.0005, MobileViT blocks:4, Transformer blocks:2, Embed dim:128 |
| Axial Transformer | Epochs: [100,150], Batch size: [32,64,128], Learning rate: [0.0001,0.0005,0.001] | Axial heads: [4,8], Axial layers: [2,4,6], Embed dim: [128,256] | 216 | Epochs:150, Batch size:64, Learning rate:0.0005, Axial heads:8, Axial layers:4, Embed dim:256 |
| ResNet-101 | Epochs: [50,100, 150], Batch size: [32,64,128], Learning rate: [0.0001,0.0005,0.001], Weight decay: [0.0001, 0.0005, 0.001], Momentum: [0.8, 0.9] | - | 162 | Epochs:100, Batch size:64, Learning rate:0.0005, Weight decay: 0.0001, Momentum: 0.8 |
| ResNet-152 | Epochs: [50,100, 150], Batch size: [32,64,128], Learning rate: [0.0001,0.0005,0.001], Weight decay: [0.0001, 0.0005, 0.001], Momentum: [0.8, 0.9] | - | 162 | Epochs:100, Batch size:64, Learning rate:0.0005, Weight decay: 0.0005, Momentum: 0.8 |
| MobileNetV3 | Epochs: [50,100, 150], Batch size: [32,64,128], Learning rate: [0.0001,0.0005,0.001], Weight decay: [0.0001, 0.0005, 0.001] | Width multiplier: [0.5,0.75,1.0] | 243 | Epochs:100, Batch size:64, Learning rate:0.001, Weight decay: 0.001, Width multiplier: 1.0 |
| CSPNet | Epochs: [50,100, 150], Batch size: [32,64,128], Learning rate: [0.0001,0.0005,0.001], Weight decay: [0.0001, 0.0005, 0.001] | CSP Block: [2,4,6] | 243 | Epochs:100, Batch size:64, Learning rate:0.0005, Weight decay: 0.0001, CSP Block: 4 |
| ConvNeXt | Epochs: [50,100, 150], Batch size: [32,64,128], Learning rate: [0.0001, 0.0005, 0.001], Weight decay: [0.0001, 0.0005, 0.001] | Stage: [tiny, small, base] | 243 | Epochs:100, Batch size:64, Learning rate:0.0005, Weight decay: 0.0005, Stage: base |

**Table S.2** Detection speed by each method on GPU (seconds per instance)

| Method | Mean | Std | Min | 25% | Median | 75% | Max |
| --- | --- | --- | --- | --- | --- | --- | --- |
| Swin Transformer | 0.0221 | 0.0007 | 0.0211 | 0.0217 | 0.022 | 0.0226 | 0.0232 |
| ViT | 0.0235 | 0.0008 | 0.0224 | 0.0229 | 0.0234 | 0.0241 | 0.0247 |
| PVT | 0.0272 | 0.0009 | 0.026 | 0.0266 | 0.0271 | 0.0278 | 0.0285 |
| MobileViT | 0.0248 | 0.0012 | 0.0233 | 0.024 | 0.0247 | 0.0256 | 0.0262 |
| Axial Transformer | 0.0239 | 0.001 | 0.0227 | 0.0232 | 0.0238 | 0.0246 | 0.0251 |
| ResNet-101 | 0.0192 | 0.0006 | 0.0184 | 0.0188 | 0.0191 | 0.0196 | 0.0203 |
| ResNet-152 | 0.0215 | 0.0007 | 0.0205 | 0.021 | 0.0214 | 0.022 | 0.0228 |
| MobileNetv3 | 0.0163 | 0.0005 | 0.0156 | 0.016 | 0.0163 | 0.0167 | 0.0171 |
| CSPNet | 0.0175 | 0.0006 | 0.0167 | 0.0172 | 0.0175 | 0.0179 | 0.0184 |
| ConvNeXt | 0.0204 | 0.0007 | 0.0196 | 0.02 | 0.0203 | 0.0208 | 0.0213 |

**Table S.3** Detection speed by each method on CPU (seconds per instance)

| Method | Mean | Std | Min | 25% | Median | 75% | Max |
| --- | --- | --- | --- | --- | --- | --- | --- |
| Swin Transformer | 0.3315 | 0.0166 | 0.2984 | 0.3203 | 0.3315 | 0.3427 | 0.3647 |
| ViT | 0.3525 | 0.0176 | 0.3172 | 0.3406 | 0.3525 | 0.3644 | 0.3877 |
| PVT | 0.408 | 0.0204 | 0.3672 | 0.3942 | 0.408 | 0.4218 | 0.4488 |
| MobileViT | 0.372 | 0.0186 | 0.3348 | 0.3594 | 0.372 | 0.3846 | 0.4092 |
| Axial Transformer | 0.3585 | 0.0179 | 0.3227 | 0.3464 | 0.3585 | 0.3706 | 0.3944 |
| ResNet-101 | 0.288 | 0.0144 | 0.2592 | 0.2783 | 0.288 | 0.2977 | 0.3168 |
| ResNet-152 | 0.3225 | 0.0161 | 0.2902 | 0.3116 | 0.3225 | 0.3334 | 0.3547 |
| MobileNetv3 | 0.2445 | 0.0122 | 0.22 | 0.2362 | 0.2445 | 0.2528 | 0.2689 |
| CSPNet | 0.2625 | 0.0131 | 0.2363 | 0.2536 | 0.2625 | 0.2714 | 0.2888 |
| ConvNeXt | 0.306 | 0.0153 | 0.2754 | 0.2957 | 0.306 | 0.3163 | 0.3366 |
